# Oxford Textbook of Philosophy and Psychiatry

**Published:** 2008

**Authors:** Ajit V. Bhide

**Affiliations:** * *Head, Department of Psychiatry, St Martha's Hospital, Bangalore - 560001, India*

“If you shut your door to all errors, the truth too will be shut out…”

…∼Rabindranath Tagore

This tome was passed on to me by a dear old classmate, now a psychiatrist in Canada, who attended the last Annual Conference of the Indian Psychiatric Society (ANCIPS 2007), with a smirk and a whisper, “Here, you might want to get started on weight lifting at least now!” Her whisper was not merely for dramatic effect; she was clearly out of breath from wielding the heavy volume, a freebie from the conference that I was unable to attend.

Weighty it is indeed, and for those who relish intellectual sparring, with insights into the *raison d'etre* of our discipline, there could be no better and closer-to-complete reference volume. The interface of the vast domains of philosophy and psychiatry is considered in five parts; the division, albeit (and even inevitably) artificial, greatly facilitates reference to particular relevant material.

The first part is “Core concepts in philosophy and mental health,” which includes some fundamentals of the two disciplines, a broad review of the Szazian antipsychiatry and its tenets, as also the arguments against these. Framed well are the usefulness and limits of the medical model. The topic of psychopathology is introduced here. Boorse's distinction between illness and disease is elaborated.

In the second part, “A philosophical history of psychopathology,” there is a succinct summary of the history of concepts of mental illness. The phenomenological approaches of Karl Jaspers and Edmund Husserl are expanded upon and the limitations of a purely phenomenological approach are rightly emphasized.

“Philosophy of science and mental health” is the third part, wherein the philosopher J.L Austin's notions of the nature of science, the place of psychiatry—and indeed of psychology—in the realm of the sciences (often so grudgingly granted by other well-recognized ‘pure’ and ‘applied’ sciences), and Freudian psychoanalysis are deliberated upon. The importance of subjective and objective judgments and the ‘evidence-base,’ much bandied about in recent times in medicine, are analyzed.

The “Values, ethics, and mental health” section, the fourth, is arguably the most useful one for the busy clinician who desires a keen and clean view on the how and why of morality in the practice of mental health.

The final section is “Philosophy of mind and mental health.” Structuralism, the Cartesian paradigm, and functionalism are expatiated upon here. Reductionism, its roots and usefulness, as well as the arguments against it, are excogitated, as are causation, freedom, rationality, and irrationality. Then there is an empathic chapter on personal identity and schizophrenia.

Beyond the five main sections, there is a concluding chapter on “Histories of the future.” This intelligent, if speculative, essay puts the whole interface in perspective, while projecting a sanguine prospect for the mental health disciplines.

## ‘Never the Twain Shall Meet?’

Shortcomings? This is a glaringly occidental volume that elaborates on the commonalities and disparities between Anglo-American and European philosophies, but takes no cognisance of the wisdom of the East at all. Even granting that psychiatry is practiced the world over on a hugely Western model, the time is more than ripe for incorporating other worldviews in “ministering to minds diseased.”

Even among philosophers of the West, I was astonished to find no mention of Carl Jung, Heinz Kohut, and Noam Chomsky. The work of E. Fuller Torrey goes unnoticed.

To my mind, no treatise that purports to be a *textbook* on the philosophy of mental processes and pathology and gives elaborate historical perspectives on these, is really complete without some elaboration on Buddhist thought and on Sankara. The reason for their conspicuous omission could not have been to keep the volume secular, for the thoughts of the Saints, Augustine and Thomas Aquinas, have merited more than a mention. I was mildly gratified to find at least a citation (unfortunately, just that) of Dinesh Bhugra's “Psychiatry and Religion”.

## Worth its Weight

This truly holistic compendium to me was an education in some respects and a rewarding revision in others. The authors, the first two apparently with a background in both the fields, do not claim to be final authorities on the ever-expanding areas covered, but profess to want to stimulate and aid the reader to contribute to the field. I do not know that we really need a *“textbook”* for this, and the book would do better without that element in its title. The case-history approach is faithfully adhered to throughout and helps sustain several arguments as well as throw up pertinent dilemmas and problems. The appended reading lists in each section are helpful.

On the whole then, a challenging and enriching collection of well-knit essays that I am sure I will repeatedly hark back to. Over-inclusion is its virtue not its bane, and Tagore's caution cited above, is thus well served. In many ways it is also a ready reckoner if that is what one is looking for; as the authors point out close to the start, you need go only to the topic you are interested in, without having to wade through a surfeit of scholarly matter of little or no immediate relevance.

My weight lifting is done for now. I do not know how good it is for muscle building, but a sharp tonic for the mind's mind, this.

## About the Author

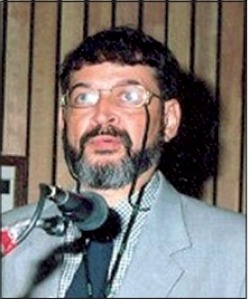



Dr. Ajit V. Bhide, heads the Departments Of Psychiatry and Family Medicine at St. Martha's Hospital, Bangalore. An alumnus of St. John's Medical College and of NIMHANS, Bangalore, he has served on the faculty of both these institutions. Dr. Bhide's main areas of interest are child and adolescent mental health, family issues in psychiatry, psychotherapy, preventive medicine, and media and mental health. He is also deeply interested in literature, history, the performing arts, and evolutionary biology.

